# Reviews of Books

**Published:** 1923-03

**Authors:** 


### REVIEWS OF BOOKS.

### MEDICINE: ANCIENT AND MODERN

**Greek Biology and Greek Medicine.** By C. Singer, M.D. (Clarendon Press; 2s. 6d. net.)

**De Arte Phisicali et de Cirurgia.** By Master John Arderne, Surgeon of Newark [A.D. 1412]. Translated by Sir D'Arcy Power, F.R.C.S. With replica of the Stockholm MS. (Wellcome Historical Medical Museum; 10s. 6d. net.)

**Arab Medicine and Surgery.** By M. W. Hilton-Simpson, B.Sc. (Oxford University Press; 10s. 6d. net.)

"That which is done is that which shall be done, and there is no new thing under the sun." Thus wrote the Preacher centuries ago, and the history of the healing art well exemplifies his saying. To all peoples and at all times disease and death have seemed grim spectres which dog the footsteps of every mortal, ready to grasp their victims. Hence the mind of man has ever striven to circumvent his enemy by all the means at his command, and the accumulated experience of what he has thought to be useful expedients has gradually acquired the fixity of tradition and custom, which, apart from direct transmission, gives a common character to the medical systems of all primitive cultures whether ancient or modern. When a real advance is made along the path of knowledge it is nearly always the work of some outstanding genius who cuts himself adrift from the intellectual hawsers which hold him, and launches out for himself. Hence the history of medicine is illustrated by a series of great names, from Aristotle and Hippocrates to Pasteur and Lister. It is not the least of the glories of Greek culture that their philosophers and physicians laid the foundations of biological and medical science so firmly that the whole structure of modern medicine has been built upon it. Dr. Charles Singer, the general editor of the series of books on the history of science, has republished his chapters on Greek medicine (which appeared in *The Legacy of Greece* a few months ago) and has added a section on Greek biology and the classificatory system introduced by Aristotle. Herein he has laid under a debt of gratitude both the zoological student and the physician, while no one can read this little book without feeling the fascination of the subject. The gigantic figure of Aristotle, metaphysician, biologist and physicist, overshadows all the science of his own day, through the Middle Ages down to our own times. Even Darwin himself acknowledges his debt. Of Greek medicine Dr. Singer writes :—

"At a certain stage in the history of the Western world men turned to explore the ancient wisdom, and the whole mass of Greek medical training was gradually laid before the student. That mass contained much dross, material which survived from early as from late Greek times which was hardly, if at all, superior to the debased compositions that circulated in the name of medicine in the middle centuries. But the recovered Greek medical writings also contained some material of the purest and most scientific type, and that material and the spirit in which it was written form the debt of modern medicine to antiquity. It is a debt the value of which cannot be exaggerated."

But it must not be supposed that medicine sprang fully grown from the brain of Hippocrates, the Father of Medicine. The Greeks had inherited a whole corpus of magical and non-rational medicine from their forerunners, which crops out again and again in Greek literature, for example, in Hesiod's "Works and Days." There is, however, abundant evidence that amongst the educated population of Athens clinical medicine was becoming a true science. Thus in his play "Philoctetes," Sophocles, though bound by the legend to attribute the sufferings of his hero to the bite of a snake, appears to have in mind the course of an attack of malaria, possibly at that time a disease only recently introduced into Greece. Similarly in his description of the madness of Ajax, Sophocles gives an extraordinarily true clinical picture of a case of acute confusional insanity. The Hippocratic oath, so often quoted, still stands to-day as a model for the ethical standard of conduct for every physician in his dealings with his patients. Not the least valuable feature of the book is the series of photographs of busts which, though probably not authentic portraits, reveal the Greek ideal of the outward form and *facies* of nobility of character and moral worth.

It is interesting to compare this description of Greek medicine with the state of medicine in England in the XIVth century shown by John Arderne's *De arte phisicali*. This has been translated from a MS. now in the Royal Library at Stockholm, which bears the date 1412. John Arderne, who was born in 1307, accompanied Henry Plantagenet as Surgeon on his travels in Europe, and possibly after his death from plague in 1364 was attached to John of Gaunt. His chief title to fame is his introduction of the cure for *fistula in ano* by cutting. The MS., which is reproduced in facsimile, is of exceptional interest by reason of the marginal illustrations and anatomical drawings. So far as medicine is concerned, the treatise shows how little advance had been made upon the Anglo-Saxon leechdoms. Sir D'Arcy Power, who has translated the manuscript, has supplied a large number of historical and explanatory notes concerning the various preparations and the names of the drugs which he recommends. Without such guidance the reader would find himself stumbling blindly amongst such terms as *Hiera picra*, *Bole armoric*, *Myrobolanus*, *Oxycrate*, and similar strange titles. The publication of the volume is due to the generosity of Mr. Henry Wellcome. The history of the MS. and how it reached Sweden is unknown No writings of Arderne have previously been published in English, a fact which makes the present volume peculiarly valuable as a contribution to the history of English medicine.

Mr. Hilton Simpson, who has spent much time in N. Africa, especially in the Aures mountains, and has made a careful study of the practice of the Shawiya native doctors, enables us to compare the medicine and surgery of Plantagenet times with those of the modern Berber inhabitants of Algeria. Certain of these native practitioners have acquired a considerable experience in the operation of trephining the skull, and in a type of bone surgery which lacks nothing in boldness and dexterity, though the instruments are primitive in the extreme. The operation of the removal of the bone is a lengthy process, but the results seem surprisingly favourable. The author gives several photographs of patients upon whom the operation had been successfully performed. But still more surprising is the frequency with which, after removal of pieces of bone from an injured limb, there has been substituted a portion of bone from a freshly killed sheep or dog. The real interest of the investigation arises from the fact that both their medicine and their pharmacopœa are derived by tradition or writings from Arab physicians of the Middle Ages, such as Ibn-el-Beitar (*c.*1220), of Malaga; Abi Nasr, who, as his name shows, hailed from Cairo (Nasr), in the same century; and Daudel-Antaki, who died in 1599. When it is remembered how potent was the influence exerted by the Arab doctors upon the progress of medicine in Europe, it is no cause for surprise to find how much there is in common between the Algerian prescriptions and those given by Arderne. To take two examples. The oily wool from the hind-quarters of a sheep is thought to have a special efficacy as a surgical dressing. Arderne used for the same purpose the " wolle that groweth atwix the legges of an ewe about the udder, full of sweat, not washed." Both use the " slough," or cast skin, of the snake as an ingredient of their medicines—a remedy also used by Thomas Sydenham. A large number of similar instances could be given.

### ENGLISH AND FOREIGN MIDWIFERY.

**Obstetrical Nursing. A Text-book on the Nursing Care of the Expectant Mother, the Woman in Labour, the Young Mother and her Baby.** By Carolyn Conant van Blarcom, R.N., formerly Assistant Superintendent and Instructor at the Johns Hospital Training School for Nurses. (Macmillan; 15s. net.)

**Obstetrics for Nurses.** By Everett D. Plass, M.D., Obstetrician-in-Chief to Henry Ford Hospital, Detroit. (D. Appleton & Co.; 15s.)

**A Text-book of Midwifery.** By R. W. Johnstone, C.B.E., M.A., M.D., F.R.C.S.E., M.R.C.P.E., Lecturer on Midwifery and Gynæcology in the School of Medicine of the Royal College of Edinburgh, etc. (A. & C. Black; 4th edition; 15s. net.)

The sub-title of " Obstetrical Nursing " gives the whole scope of its contents, but there is no mention of midwifery, as it is understood in England. Being written by an American author, it must be remembered that in the United States and Canada midwives are not recognised as such, but work always under the direction of the doctor. Nevertheless, the book may be studied with exceedingly helpful results by British midwives, for while not representing the views of any one doctor, or one school, it is written on lines broad enough to embrace the underlying principles of good obstetrical nursing everywhere. It is interesting to compare the methods of one country with another in this important branch of nursing, though the " general principles of cleanliness, watchfulness, adaptability and sympathetic understanding will apply to the nursing of all patients." The author ordinarily allows the normal mother to sit up in bed about the sixth or eighth day. In England sitting up is generally allowed after two or three days, and in Austria still earlier, for in the *Frauen-Hospiz* (Vienna), after 24 hours of lying flat in bed, the patient is propped up for a while, gets up on the third day, and goes home on the eighth. Miss Van Blarcom has a pleasant style and her book is eminently readable, as well as teeming with useful information. The chapter on the nursing care of the average new-born baby contains exhaustive directions as to correct infant feeding. A few words at the end summarise the whole very completely. The author concludes by saying that, " on the whole, the care of all babies the year round resolves itself into the observation of a few general principles, namely: proper feeding; fresh air; regularity in his daily routine; cleanliness of food, clothing and surroundings; maintenance of an equable body temperature and conservation of his forces." There are 200 excellent illustrations. We recommend this book to midwives and nurses.

In " Obstetrics for Nurses " the author, who is an American obstetrician, has succeeded very well in attaining his avowed object of condensing and incorporating the essence of the knowledge of obstetrics without the presence of the confusing mass of detail found in similar works for the medical student. Primarily addressed to obstetric nurses with general training, who are therefore presumably acquainted with the usual technique of surgical asepsis, detailed accounts of this part of the midwifery nurse's work are not included in its pages. The book is rather a " simplified treatise on the more or less scientific side of this specialty."

It would, however, at the same time, especially the earlier chapters, be equally appropriate for study by the prospective mother herself, or by her friends, as such matters are discussed as are of common interest to all—for instance, normal pregnancy and pre-natal care; the physiology and mechanism of labour; also the useful chapter on the normal child and its care. In this connection the author's views on the bathing of new-born infants are of interest. Until the cord has separated, and the wound healed, he recommends, instead of bathing, the use of " oil rubs." This procedure, he says, has long been advocated in the case of premature infants, because it tends to conserve the body heat, and the same argument supports the treatment in mature children. He adds, " There is, moreover, a considerable saving of time." Four-hourly breast-feedings are recommended for healthy infants. The " pacifier " (comforter) is condemned. The second half of the book deals with diseased and abnormal conditions during pregnancy, labour, the puerperium, and in the new-born. It also describes certain obstetrical operations, beginning with the simple procedures of douching and " packing " to those which facilitate delivery, and are usually of an " emergency " character.

A large number of excellent photographs and drawings illustrate the letterpress through the book, and the index is particularly full and clear.

" A Text-Book of Midwifery " was first published in 1913, and a third edition appeared in 1920. That there should so quickly be a demand for a fourth shows in the most practical manner how the interest first awakened in this excellent manual has been sustained and is rapidly increasing. Written for students and practitioners, it is also widely used as a text-book for pupil-midwives, and, being very clearly expressed, is, for the most part, within the mental grasp of such as have not received the advantages of a first-class education. The author has aimed at making the book thoroughly practical and up to date, and in the present edition are several alterations and much new matter. The chapter on the Duration and Hygiene of Pregnancy, which contains a new section on Ante-Natal Supervision, should be valuable to those who are establishing or working at ante-natal clinics.

### GROWING OLD GRACEFULLY.

**Some Medical Aspects of Old Age.** By Sir Humphry Rolleston, M.D., P.R.C.P. (The Linacre Lecture, 1922.) (Macmillan; 6s. net.)

Old age has a universal interest, and the discovery of the elixir of life has ever been the hope of mankind. The uncertainty of life, and the natural dread of death, with its end to our human joys and fears, have given to old age a peculiar glamour. For the young, as life unfolds before them, it is a goal which each hopes to attain; for those who have reached the allotted span of life " the long habit of living indisposeth us for dying," and old and young alike re-echo the old refrain, "*Timor mortis conturbat me.*" Yet the medical aspects of old age have received surprisingly little consideration. Sir Humphry Rolleston has taken advantage of the opportunity presented by the Linacre Lecture at St. John's College, Cambridge, to re-survey the field in the light of modern knowledge. This lectureship was founded in 1524 by Thomas Linacre, the first President of the Royal College of Physicians, who, though an Oxford man himself, generously created a lectureship at both Universities. It was, therefore, peculiarly fitting that the choice of a Linacre lecturer should again fall upon the President of that Royal foundation, a double distinction shared with Sir Thomas Watson (President, 1862-67) and Sir Norman Moore, his immediate predecessor in the Presidential chair, and with others whose names are less well known to us to-day.

In defending his choice of a subject, Sir Humphry Rolleston aptly quotes the words of Descartes—" We might be free from an infinity of maladies, both of body and mind, if we had sufficient knowledge of their causes and remedies." Old age comes so gently and imperceptibly that those who have attained to it tell us that they have never felt old. Sir George Humphry, who published a very notable study of age, with an analysis of a large number of cases, used to say that no one was really " old " till he was eighty, an age which he himself never reached. The author quotes evidence to show that one of the most potent factors in longevity is heredity. This is a general experience, and, indeed, " nature " seems in this case to be able largely to circumvent the effects of an adverse environment, but there is no doubt that a wise management of health may do much towards a prolongation of life. A healthy regimen seems to be more valuable in this connection than the alleged rejuvenescence through the medium of one or other of the endocrine glands. The view urged by Metchnikoff that senescence is due to the deleterious action of the products of the intestinal flora upon the tissues of the body, which can be inhibited by the ingestion of appropriate bacteria, such as *Bacillus Bulgaricus*, seems to have something in its favour besides its simplicity. It fails, however, wholly to satisfy the rigid conditions of experience, though there are many oracular sayings which describe the influence of the alimentary canal. Montaigne says, concerning the addiction to the pleasures of the table, " Man does not die, he kills himself."

The author touches upon a most interesting subject when he raises the question of the influence of auto- and hetero-suggestion. He quotes Finot as stating that without the malignant influence of suggestion man might live to 150 years! As Ibsen taught in, the *Master Builder*, the younger generation is ever pressing upon the heels of its elders, and the suggestion, whether it comes from within or without, that he has " had his day " may readily bring to a man that loss of self-reliance which, as Halvard Solness feared, is a prelude to a cessation of the struggle and of the " will to live." The potency of this lowered mental activity in the induction of senility is seen, according to Thompson and Todd, in the case of the Chelsea Pensioners. These observers urge that it may be reduced by cheerful badinage and the avoidance of too much sympathetic condolence. True it is that those who have continued in the active pursuit of their profession tend to live to a greater age than those who retire to a life of leisure, which all too readily degenerates into a self-centred apathy.

No book on old age could be considered complete without some reference to the beautiful chapter in the Book of Ecclesiastes, and the author devotes an interesting section to the various interpretations which have been placed upon the metaphors which it contains. The whole lecture is a scholarly production, worthy alike of its occasion, the intrinsic interest of the subject, and the pre-eminent position in the medical profession occupied by its author.

### MISCELLANEOUS NOTICES.

### Physics as an Educator.

**Practical Physics.** By J. A. Crowther, D.Sc. Hodder & Stoughton. 10s. 6d. net.—This book has been designed to cover the practical work in physics which is required of a student in the first medical examination. It has been compiled with great care, and bears the mark of its author's practical experience. He regards his subject as a useful method of inculcating habits of accuracy and thoroughness, and certainly any student who works through and masters the book will not only have learnt enough to satisfy his examiners, but will have acquired habits of accurate observation and trustworthy record making which will stand him in good stead throughout his subsequent career.

### In Aid of the Blind.

The Rainbow: A Drama of Light. By Gertrude H. Witherby. Grant Richards. 3s. 6d. net.—This little allegorical play is written by one of the most devoted helpers at St. Dunstan's, and contains a preface by the late Sir Arthur Pearson. "I hope," he wrote, "that the readers of this little book may find therein some comfort for their own sorrows, and that a new ray of light on the eternal problem of suffering and sacrifice may illumine hours of darkness and pain. If this should prove to be the case, will they remember the needs of our blinded soldiers and sailors, whose path in life has been darkened in order that the world may gain in Freedom and Light ?" All the author's profits on the sale of the book will be given to the Blinded Soldiers' and Sailors' After-Care Organisation.

### First Aid and X-Rays.

First Aid X-Ray Atlas of Fractures and Dislocations. By H. C. Orrin, O.B.E., F.R.C.S.E. Baillière, Tindall & Cox. 1922.—This handy little book contains a series of radiograms of the various fractures and dislocations of the bones, together with photographs of the bones in the normal state. It should be of real value to the student and teacher of first-aid and ambulance, since it shows also the correct methods, photographed, of applying splints and bandages appropriate to each case.

### Chemical Apparatus.

Modern Chemical Lecture Diagrams. By Geoffrey Martin, D.Sc., Lond. and Brist., Ph.D., F.I.C., F.C.S. Assisted by J. M. Dickson, B.Sc., and Major J. W. Christelow, B.Sc. With 36 original diagrams. Sampson Low, Marston & Co.; 3s. 6d. net.—The illustrations are of complicated apparatus or industrial processes usually treated of in courses on chemistry. On the score of expense it is obvious that only a few institutions could afford the plant or apparatus employed, for instance, in the manufacture of nitric acid and nitrates from the atmosphere, or in the making of calcium carbide by the continuous furnace. Each diagram figured in the book may be obtained greatly enlarged in chart form, which will be found of great assistance to lecturers in explaining the more advanced processes of chemistry to their classes.

### Medicine in the Mirror.

A Synopsis of Medicine. By H. Letheby Tidy, M.A., M.D., B.Ch., F.R.C.P. John Wright & Sons, Bristol. Third Edition. 21s. net.—A bird's-eye view of the whole of medicine in the shape of a condensed account of the diseases of the various organs and systems is of great value to the student, and the practitioner. In Dr. Tidy's now well-known synopsis the opportunity has been taken in this, the third, edition to revise the whole work, and to incorporate much new matter. About thirty pages have actually been added, and these comprise information upon the physiology of digestion, cyclical vomiting, cœliac disease, cocaine and veronal poisoning, sprue, and the newer tests of renal efficiency. The value of insulin in diabetes, and of fractional test meals, are referred to in the appendix. The copious index is not the least valuable feature of this comprehensive work, which will be prized alike by the student and the busy practitioner.

### The Future of the Settlement.

Settlements and Their Outlook. P. S. King. 2s. 6d. net.—This is an account of the first International Conference of Settlements, which was held at Toynbee Hall last July. It has not been possible to print the papers in full, nor was a little volume of summaries and impressions thought adequate, so a compromise has been attempted, and lengthy quotations made from the papers, the setting and atmosphere of these being preserved by a certain amount of description and comment. The Conference revealed an unsuspected extent and variety in settlement work throughout the world and established the necessity of developing in every country a strong national movement. "But it made inevitable some provision for the development, as a result, of a world movement." Continuation Committees have therefore been set up in each co-operating country, and another International Conference will be arranged for 1925.

### A Hospital Pharmacopœia.

Pharmacopœia of the Royal Victoria Infirmary, Newcastle-on-Tyne.—This convenient little book, besides containing all the matter usually found in hospital pharmacopœias, has the advantage that doses are given in English and metric measures, and that notes upon incompatibility, solubility, &c., are given under the headings of each drug. Brief descriptions of food preparation, food values and methods of simple chemical and bacteriological examination are given. It is a handy little volume of reference for the pocket or desk.

### Physiology and Medical Practice.

The New Physiology in Surgical and General Practice. By A. Rendle Short, M.D., B.S., B.Sc. John Wright & Sons, Bristol. Fifth Edition. 9s. 6d. net.—To keep up with the advances made in physiological research and their consequent bearing upon the every-day problems of practical medicine and surgery a fifth edition of Professor Short's illuminating book has been called for. In addition to a complete revision of the whole work, three fresh chapters have been added, dealing with the functions of the kidney, the dietetic factor in the causation of appendicitis, and the physiology of muscular exercise. Dr. C. E. K. Herapath has also rewritten the chapter on the heart. The interesting suggestion is thrown out that the increase in appendicitis in this country coincided with the beginning of the importation of foreign food and the diminished national consumption of cellulose-containing foods. An added zest for the day's work may be obtained by reading this stimulating book.

### A Hospital Annual.

Burdett's Hospitals and Charities. The Scientific Press. 17s. 6d.—This is the thirty-third year of issue of an invaluable book of reference. The same plan of condensation and revision has been carried out as in the 1920 and 1921 editions, and the "Directory of Institutions" has been further drastically overhauled. The policy has been, as stated in the preface, "to lop off dead branches from the tree" and the exclusion of all obsolete information and information that cannot be verified has made room for certain additional matter which should be distinctly useful. The military and naval section has been augmented. Hospitals where the Ministry of Pensions has financial liability have been added, the new names of Poor Law infirmaries are given and a list of the Local Committees set up by the Voluntary Hospitals Commission appointed by the Ministry of Health follows the preliminary chapters. The tables of income and expenditure of convalescent homes which were omitted last year have been reinstated and the list of consumption sanatoria amplified. Thoroughly trustworthy and up-to-date in its information, the new edition well maintains the reputation of this excellent annual.

### A Book for Young Mothers.

Common Sense in the Nursery. By Charis Barnett, M.A. Christopher. 6s. net.—This is a simple unpretentious little book containing some sound advice to young mothers. It is concerned not only with the physical health of the child, but with its general management—its toys and books, its manners and its moral welfare. There is even a chapter of "advice to strangers"—much needed advice, too, it is—on the best means of making friends with children. "Most babies like men best," because they leave a child alone at first, while "women, who should know better, nearly always rush at a baby, often envelop him, and snatch him up before he realises what is happening." An appendix provides some useful recipes for appetising dishes, and another deals with baby shows. "Health workers warn us that advertised baby shows are altogether bad, as the mothers tend to fatten up the baby at the expense of the rest of the family. . . . But baby shows at bazaars and similar functions that are not advertised for long beforehand are not open to this objection and have their uses." The author's final word is worth attention: "Children should be, of all the people in the house, the most important and the most seriously considered, but they should think that less attention is paid to them and to their likes and dislikes than to any other member of the household."

